# Supplementary material for: New TMA (4,6,4′-Trimethyl angelicin) Analogues as Anti-Inflammatory Agents in the Treatment of Cystic Fibrosis Lung Disease
Source: Int J Mol Sci. 2022 Nov 21;23(22):14483. doi: 10.3390/ijms232214483 (PMC9699093; doi:10.3390/ijms232214483)
Supplement: Supplementary file 1 [file ijms-23-14483-s001.zip › ijms-1967167-supplementary.pdf]

Supplementary material

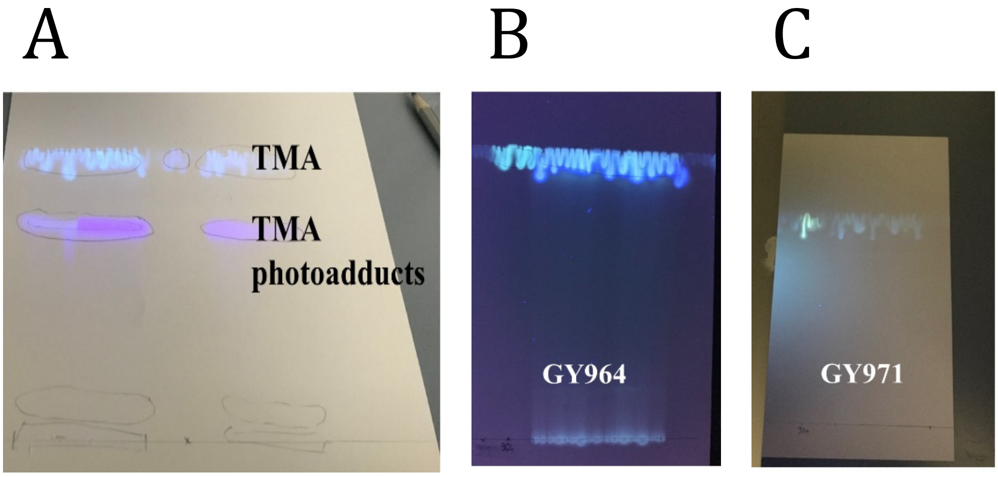

Figure S1. TLC of TMA (A), GY964 (B) and GY971 (C). Image analysis under UV irradiation to evaluate photoadduction (pictures extracted by Vaccarin, C.; Gabbia, D.; De Martin, S.; Franceschinis, E.; Roverso, M.; Bogialli, S.; Sacchetti, G.; Tupini, C.; **Lampronti, I**.; Gambari, G.; Cabrini, G.; Dechecchi, M.C.; Tamanini, A.; Marzaro, G.; Chilin, A. Improved Trimethylangelicin Analogues for Cystic Fibrosis: design, synthesis and preliminary screening, ***Int J Mol Sci***, *2022* 23, 115028).

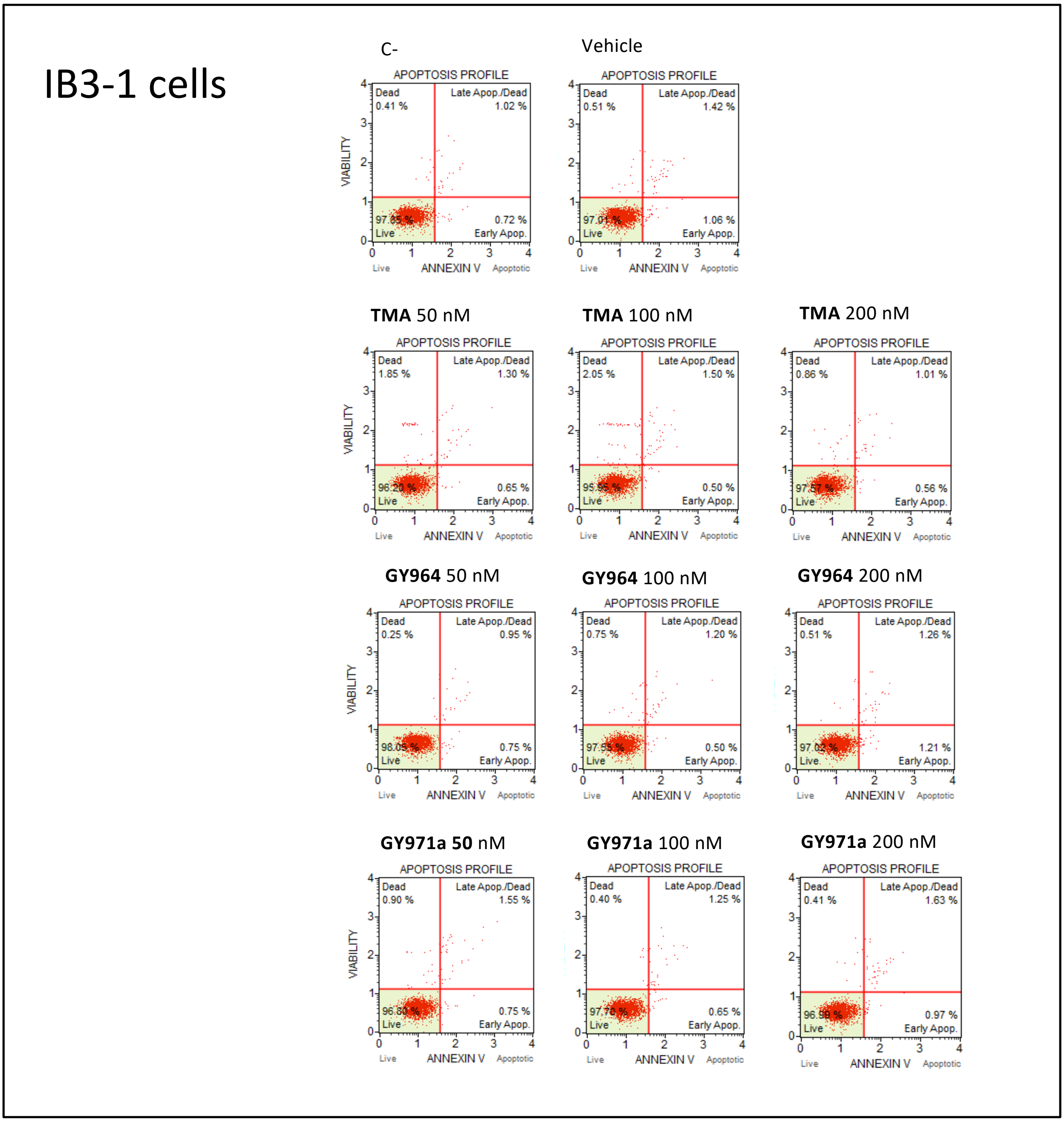

Figure S2. Annexin V assay on IB3-1 cells untreated (C-) and treated with DMSO (Vehicle) and 50, 100, 200 nM TMA, GY964 and GY971a.

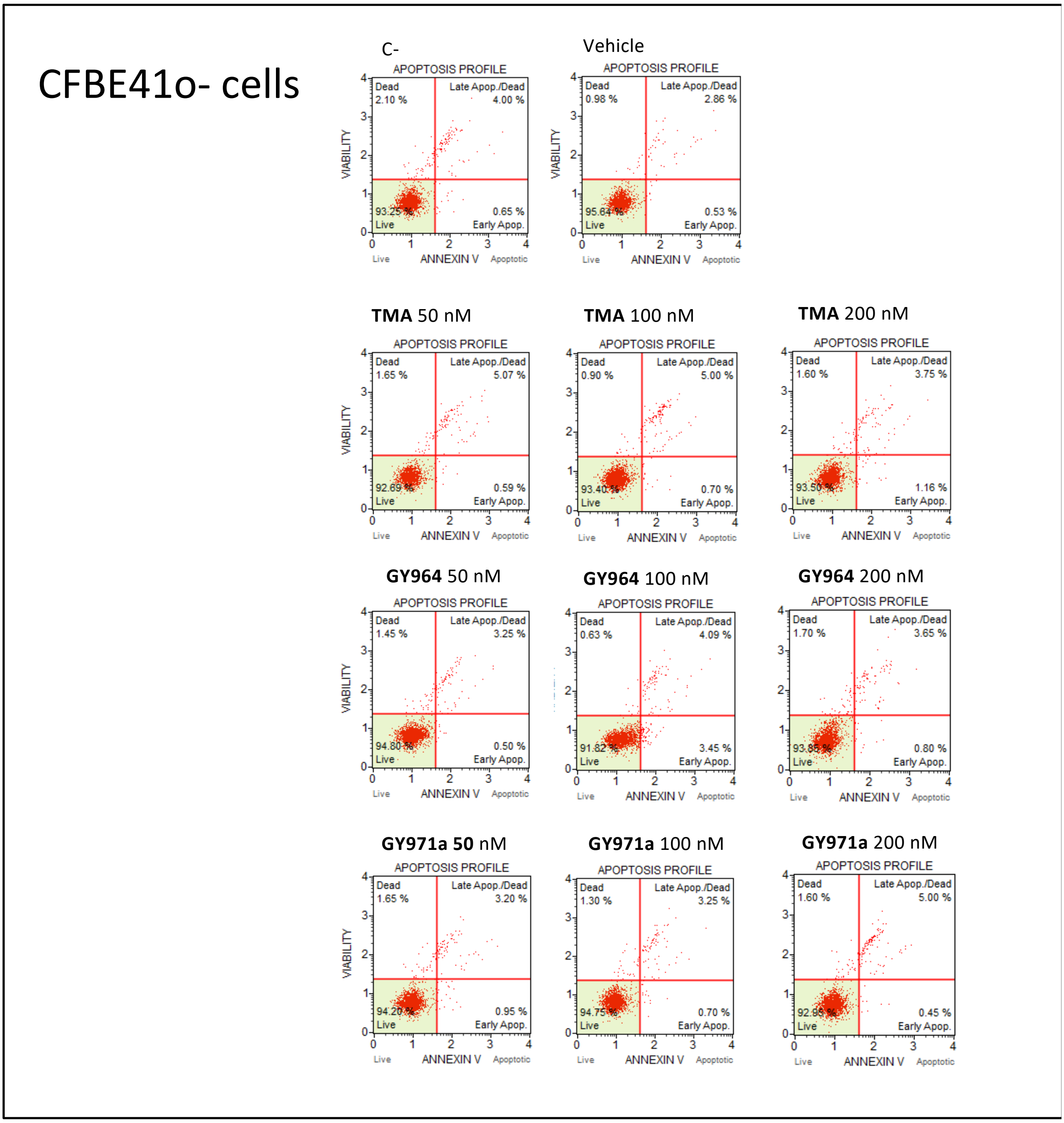

Figure S3. Annexin V assay on CFBE41o- cells untreated (C-) and treated with DMSO (Vehicle) and 50, 100, 200 nM TMA, GY964 and GY971a.
